# Diabetes mellitus: Preventive and curative therapies with aqueous extract of *Rytigynia senegalensis* Blume (Rubiaceae) in Wistar rats

**DOI:** 10.1016/j.jtcme.2023.03.001

**Published:** 2023-03-01

**Authors:** Barthelemy Maidadi, Fidèle Ntchapda, David Miaffo, Abba Talba Mahamad

**Affiliations:** a Laboratory of Animal Physiology and Pharmacognosy, Department of Biological Sciences, Faculty of Sciences, University of Maroua, P.O. Box 814, Maroua, Cameroon; b Laboratory of Medicinal Plants, Health and Galenic Formulation, Department of Biological Sciences, Faculty of Sciences, University of Ngaoundere, P.O. Box 454, Ngaoundere, Cameroon; c Laboratory of Life and Earth Sciences, Department of Life and Earth Sciences, Higher Teachers' Training College, University of Maroua, P.O. Box 55, Maroua, Cameroon

**Keywords:** Diabetes mellitus, *Rytigynia senegalensis*, Streptozotocin, Lipid profile, Antioxidant

## Abstract

**Background and aim:**

African traditional healers use *Rytigynia senegalensis* Blume to treat diseases such as diabetes mellitus, malaria, dysentery, constipation, and hemorrhoids. This study aimed to assess the hypoglycemic, lipid-lowering, and antioxidant properties of *R. senegalensis* extract (AERS) in type 1 diabetic (T1D) and insulin-resistant (T2D) rats.

**Experimental procedure:**

The induction of T1D was made by intraperitoneal administration of streptozotocin (55 mg/kg b.w). As for T2D, it was induced for 10 days by daily subcutaneous administration of dexamethasone (1 mg/kg b.w). Diabetic animals were divided and treated with AERS (50, 100, and 200 mg/kg b.w) for 28 and 10 days for T1D and T2D, respectively. Glycaemia, food and water consumption, relative body weight, insulinemia, lipid profile, and oxidative stress parameters were evaluated. Histological sections were made on the pancreas of T1D rats.

**Results and conclusion:**

AERS (100 and/or 200 mg/kg) prevented (p < 0.05 to p < 0.001) weight loss, polyphagia, and polysipsia in diabetic rats. AERS significantly lowered (p < 0.05 to p < 0.001) insulinemia, hyperglycemia, triglycerides (TG), low-density lipoprotein cholesterol (LDL-c) total cholesterol (TC),and malondialdehyde (MDA). In contrast, a significant increase (p < 0.05 to p < 0.001) in high-density lipoprotein cholesterol (HDL-c) levels, reduced glutathione levels, and superoxide dismutase (SOD) and catalase (CAT) activities were observed with all doses of AERS. Histopathological analysis showed an increase in the number and size of islets of Langerhans in the pancreas of T1D rats receiving AERS. AERS has an important antidiabetic, antidyslipidemic, and antioxidant potential.

## Highlights

• *Rytigynia senegalensis* has antihyperglycemic and hypoglycemic properties, and therefore antidiabetic.
• Treatment with *Rytigynia senegalensis* prevents or restores oxidative damage to organs.
• *Rytigynia senegalensis* possess anti-dyslipidemia and cardioprotective properties.
• These observed pharmacological effects would be due to the bioactive constituents present in *Rytigynia senegalensis*.

## Graphical abstract

**Figure:**
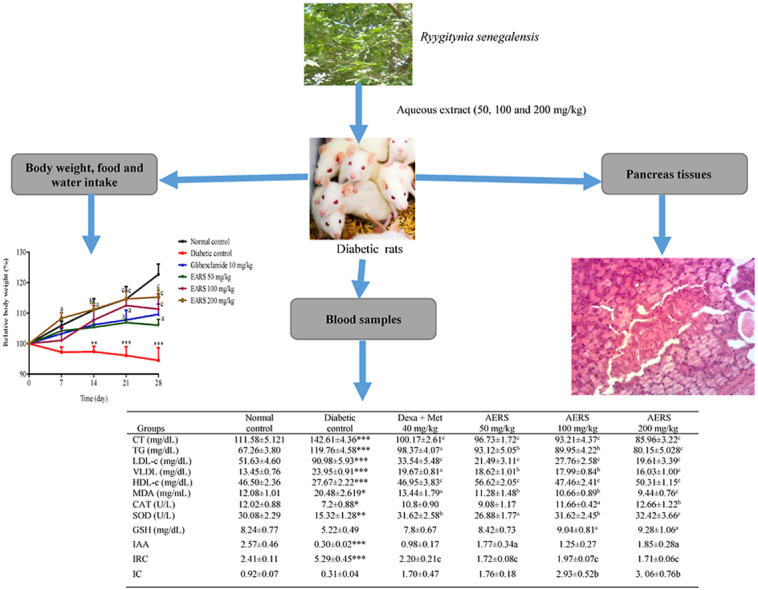

## List of abbreviations

EARS: Aqueous extract of *Rytigynia senegalensis*
T1D: Type 1 diabetic
T2D: Insulin-resistant
TG: Triglycerides
LDL-c: Low-density lipoprotein cholesterol
TC: Total cholesterol
MDA: Malondiadhyde
HDL-c: High-density lipoprotein cholesterol
SOD: Superoxide dismutase
CAT: Catalase
WHO: World Health Organization
ELISA: Enzyme Linked ImmunoSorbent *Assay*
STZ: Streptozotocin
*HOMA-β*: Homeostasis model assessment of β-cell function
HOMA-IR: Homeostatic model assessment of insulin resistance
VLDL-c: Very low-density lipoprotein cholesterol
AAI: Anti-atherogenic index
CRI: Coronary artery risk index
CI: Cardioprotective index
SEM: Standard error of the mean
AMPK: Adenosine monophosphate kinase

## 1 Introduction

Diabetes mellitus is a metabolic disease characterized by the inability of the body to produce or respond to the action of insulin.^1^ It is a state of chronic hyperglycemia that appears when blood sugar is greater than or equal to 1.26 g/L.^2^ Untreated hyperglycemia can lead to complications in the eyes, kidneys, nerves, heart, and blood vessels.

There are two forms of diabetes mellitus: type 1 diabetes (T1D) and type 2 diabetes (T2D). T1D is characterized by the destruction of β cells in the pancreas; it most often occurs before the age of 20 and represents 10–15% of diabetes. Type 2 diabetes is characterized by resistance of the liver, adipose tissue, and the muscles to the action of insulin; it usually occurs after the age of 50 and accounts for 85–90% of diabetes.^3^ Several studies suggest that diabetes is accompanied by oxidative stress which disrupts insulin resistance and promotes the cardiovascular complications associated with it. About 422 million people had diabetes in 2016, and by 2040, 642 million people will have it if left unchecked.^4^ The global death rate from diabetes mellitus was 1.6 million in 2015 and 4 million in 2017.^5^^,^^6^

The treatment of diabetes is mainly based on the administration of oral antidiabetics and the injection of insulin. Not all of these anti-diabetic agents meet the needs of patients as an effective treatment and eventually side effects are observed. This is a state of hypoglycemia, digestive problems, cardiovascular risks, weight loss due to strict diet.^7^ The infrastructure and health personnel are insufficient. Conventional medicine is only accessible to a privileged category of the population. Some conventional drugs as well as hospital costs are expensive and their supply remains insufficient.^8^ Under these conditions, more than 80% of our populations have resorted to traditional medicine since ancient times. The potential richness of traditional medicine all over the world has prompted WHO to develop a strategy for the development and evaluation of this medicine.^9^ Many researchers have turned to the ethnopharmacological approach to find new bioactive compounds, to develop improved traditional drugs.

*Rytigynia senegalensis* Blume a plant in the Rubiaceae family and to treat diabetes mellitus, malaria, dysentery, constipation, and hemorrhoids. It is native to West Africa, it has a very diverse geographical origin, most of it is found in the Sahelian zone (Senegal, Ivory Coast, Sudan and Cameroon).^10^ It commonly known as the ‘‘rytigyne du Senegal’’ and also called Delbi in the northern part of Cameroon. However, no scientific study has already proven the antidiabetic effect of *R. senegalensis*. The present study aimed to assess the hypoglycemic, lipid-lowering, and antioxidant properties of AERS in T1D and T2D rats.

## 2 Materials and methods

### 2.1 Chemicals and reagents

Dexamethasone, glibenclamide, metformin, potassium dichromate, ketamine, and diazepam were bought at the pharmacy in the Far North, Cameroon. Streptozotocin (STZ), rat insulin ELISA, and biochemical kits were purchased from Sigma-Aldrich, Saint. Louis, USA. Sodium chloride and d-glucose were provided by the Edu-Lab Biology Kit, Bexwell, UK. Reagents and chemicals used in this study were obtained commercially in analytical grade.

### 2.2 Preparation of the plant extract

*Rytigynia senegalensis* was collected in the locality of Pette, a village located 70 km from the city of Maroua, Cameroon. A sample of it was identified by the botanist Dr. Todou Gilbert and kept at the National Herbarium (Cameroon) under number 4496/SRFK. The harvested plant was washed with tap water, dried at room temperature out of the sun, and reduced to a fine powder.

A mass of 14.28 g of the powder obtained was introduced into 500 mL of distilled water previously boiled for 30 min. After cooling, the infusion was filtered using Whattman No. 1 paper. The resulting liquid was dried in an oven set at 45 °C for 48 h to obtain the crude extract of *R. senegalensis* (mass = 1.01 g; yield = 7.07%).

### 2.3 Qualitative phytochemical screening

Phytochemical screening is a qualitative analysis based on precipitation and/or color reactions. This test allowed us to highlight the different classes of bioactive compounds such as quinones (NH_4_OH), alkaloids (Mayer's reagent), flavonoids (HCl and Mg), saponins (stable foam), tannins (FeCl_3_), sterols (acetic anhydride), and phenols (FeCl_3_ and K_3_Fe(CN)_6_).^11^

### 2.4 Animals

Animals consisted of male rats of the Wistar strain, aged between 10 and 12 weeks, and weighing between 220 and 250 g. They were provided by the Laboratory of Medicinal Plants, Health and Galenic Formulation of the University of Ngaoundere (Cameroon) in polystyrene cages under conditions of ambient temperature (25 ± 2 °C) and natural light (12h light-dark cycle). Drinking water and food were given *ad libitum* for the duration of the experiment. The acclimatization of the animals lasted 2 weeks before the start of each test. The experimental animals were handled in accordance with the Cameroon National Ethics Committee (Ref. N ° FWIRB 00001954), and internationally accepted principles for the use and care of laboratory animals as contained in the Community guidelines European (EEC Directive 1986; 86/609/EEC).

### 2.5 Induction of T1D and treatment of animals

To induce T1D, a solution of STZ (diluted in a citrate buffer 0.1 mol/L, pH 4.5) at a dose of 55 mg/kg was administered intraperitoneally in the rats previously fasted for 14 h. To avoid hypoglycemic shock, d-glucose solution (5%) was administered orally to all animals. Three days after the STZ injection, the blood glucose levels of the rats were assessed and those with blood glucose greater than or equal to 200 mg/kg were selected for the experiments.^12^

Thirty animals (5 normal and 25 diabetic rats) were randomly divided into 6 groups (n = 5) and treated orally for 28 days. Groups I (normal control) and II (diabetic control) received 10 mL/kg b.w of distilled water. Group III (standard control) received glibenclamide (10 mg/kg b.w). Groups 4, 5, and 6 received AERS at doses of 50, 100, and 200 mg/kg b.w, respectively. Fasting blood glucose, body weight, water consumption, and food intake were assessed on days 0, 7, 14, 21, and 28 of treatment.

### 2.6 Induction of T2D and treatment of animals

Thirty (30) male animals were divided into 6 groups (n = 5) and treated orally daily for 10 days as follows: groups I (normal control) and II (diabetic control) received 10 mL/kg b.w of distilled water; group III (standard control) received metformin (40 mg/kg b.w), and groups 4, 5, and 6 received AERS at 50, 100, and 200 mg/kg b.w respectively. One hour after these different treatments, all animals received a dexamethasone solution (1 mg/kg b.w) subcutaneously, except the rats of the first group which received by the same route the solution of NaCl 0.9% (1 mL/kg b.w). Body weight, glycaemia, water intake, and food consumption were assessed on days 0, 5, and 10 of the experiment.

### 2.7 Blood and pancreas samples

Immediately after the end of the treatments, the animals were fasted for 24 h and then anesthetized with ketamine (50 mg/kg b.w) and diazepam (10 mg/kg b.w). The abdominal cavity was opened and blood was drawn by cardiac puncture, put into dry tubes and centrifuged at 3000 rpm for 15 min. The supernatant collected was kept at −20 °C for assaying insulin, lipid profile, and oxidative stress parameters.

### 2.8 Assessment of biochemical parameters

Fasting blood glucose was measured using a One Touch Ultra Mini glucometer and strips. Serum insulin level was evaluated by the method of Herbert et al.^13^ with the ELISA kit. Homeostatic model assessment (HOMA-β and HOMA-IR) scores was calculated using fasting blood glucose and fasting insulin levels.^14^ HOMA-β = 20 × fasting insulin (U/L)/fasting glucose (mmol/L) - 3.5. HOMA-IR = fasting glucose (mmol/L) (U/L) × fasting insulin/22.5.

Total cholesterol (TC) level was assayed according the enzymatic colorimetric method described by Trinder.^15^ High-density lipoprotein cholesterol (HDL-c) level was determined by the method of Weibe et al.^16^ The determination of triglycerides (TG) level was carried out according to the enzymatic colorimetry method.^17^ Low-density lipoprotein cholesterol (LDL-c) level was deduced from the other lipids.^18^ The formula described by Friedewald et al.^19^ was used to calculate the very low-density lipoprotein cholesterol (VLDL-c) level. Anti-atherogenic index (AAI) was calculated as AAI = Log (TG/HDL-c).^20^ Coronary artery risk index (CRI) was calculated using the formula described by Barter et al.^21^: CRI = TC/HDL-c. The formula described by Jukema et al.^22^ was used to calculate the cardioprotective index (CI) value: CI = LDL-c/HDL-c.

Malondialdehyde (MDA) level was evaluated by the method described by Wilbur et al.^23^ with slight modifications. The methods of Sinha^24^ and Kakkar^25^ were used to determine the catalase (CAT) and superoxide dismutase (SOD) activities respectively. Reduced glutathione (GSH) level was evaluated according to the method of Ellman.^26^

### 2.9 Histological analysis of the pancreas

The pancreatic tissues of T1D rats fixed in a 10% formaldehyde solution were dehydrated in a graduated series of alcohol incorporated into paraffin blocks. A semi-automated rotator microtome allowed us to prepare 4 μm thick sections of pancreatic tissues. The tissue sections were then mounted on glass slides, then deparafinized by xylene and rehydrated by different graded ethanol dilution (100%, 90%, and 70%). The sections were stained with hematoxylin and eosin (H&E) and all slides were observed using light microscope and photographed at 100X magnification.

### 2.10 Statistical analysis methods

Data were expressed as the mean ± Standard Error of the Mean (SEM) and analyzed with the Graph Pad Prism software version 5.0. One-way analysis of variance and Turkey's posttest were used to analyze data from the single-variable test. Two-way analysis of variance and Bonferroni's posttest were used for the treatment of double-variable tests. The differences were considered significant at the probability threshold of 0.05.

## 3 Results

### 3.1 Phytochemical screening

Secondary metabolites such as flavonoids, phenols, tannins, alkaloids, terpenoids, coumarins, glycosides, and anthraquinones were present in AERS. In contrast, quinones, saponins, and sterols were absent in AERS. .

### 3.2 Effects of AERS on relative body weight, water intake, and food intake in T1D animals

**Fig. 1:**
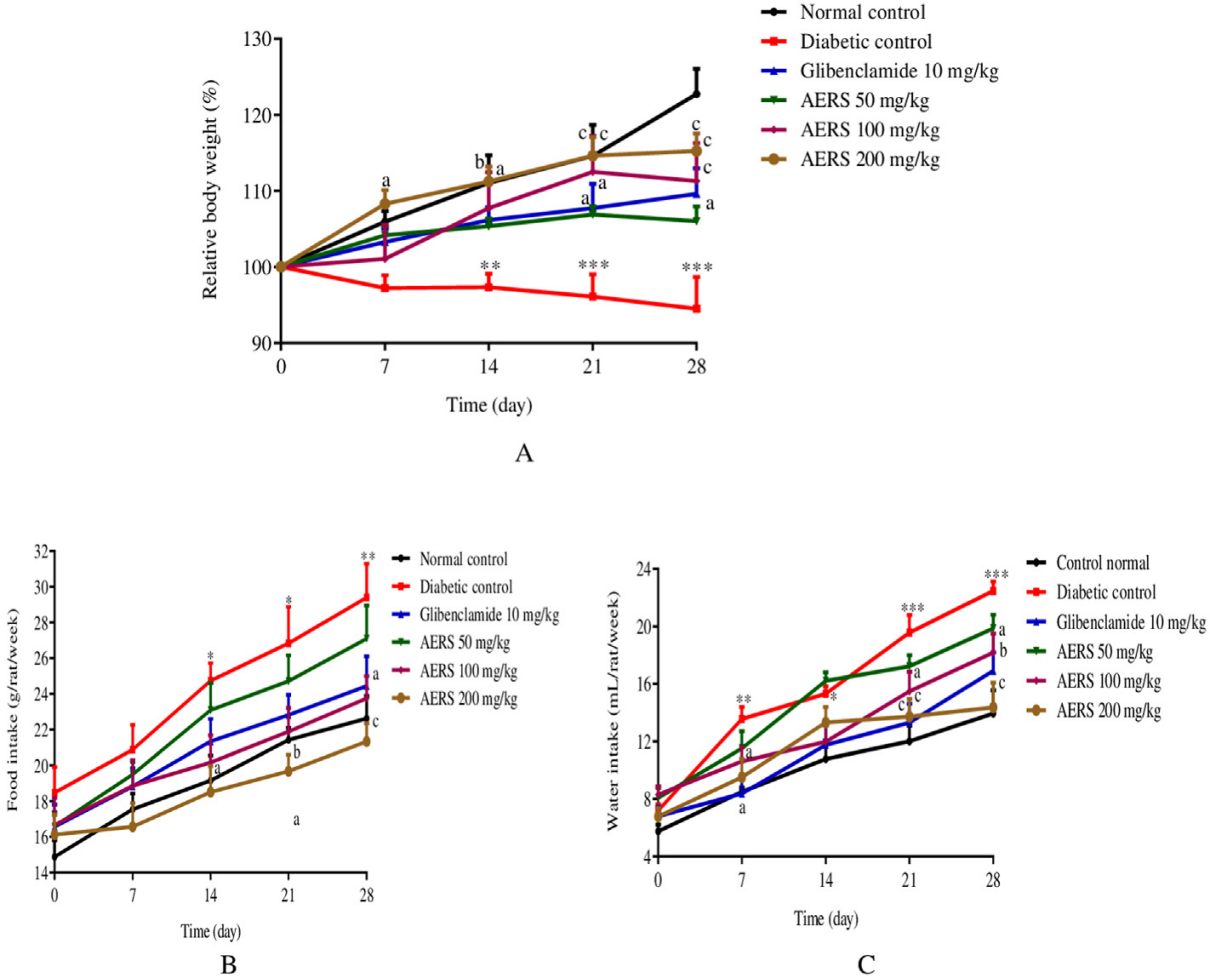
Effects of AERS (50, 100, 200 mg/kg) and glibenclamide (10 mg/kg) on relative body weight (A), food consumption (B), water intake (C) in streptozotocin-induced T1D rats. Data were expressed in mean ± S.E.M. (n = 5). ∗p < 0.05; ∗∗p < 0.01; ∗∗∗p < 0.001 compared to the normal control group. ^a^p < 0.05; ^b^p < 0.01; ^c^p < 0.001 compared to the diabetic control group.

Fig. 1 shows the effects of different treatments on the relative body weight (A), and food (B) and water (C) intake of T1D rats. It emerges from this figure that in the diabetic control group, there was a decrease in relative body weight on days 14 (p < 0.01), 21 (p < 0.001), and 28 (p < 0.001) of the experiment, compared to the normal control group. However, animals receiving AERS at 200 mg/kg showed a significant increase in their relative body weight on days 7 (p < 0.05), 14 (p < 0.01), 21 (p < 0.001), and 28 (p < 0.001) of treatment, compared to the diabetic control group (Fig. 1A). Likewise, the body weight of animals receiving glibenclamide and AERS (100 mg/kg) increased on days 14 (p < 0.05), 21 (p < 0.01), and 28 (p < 0.001).

In the diabetic control group, food intake increased (p < 0.05) on days 14, 21, and 28, compared to the normal control group. However, at the 28th day of treatment, a significant drop in food consumption was noted at 100 mg/kg of AERS, compared to the diabetic control group. AERS (200 mg/kg) significantly reduced food consumption on days 14 (p < 0.05), 21 (p < 0.05), and 28 (p < 0.01) (Fig. 1B).

In diabetic control group rats, an important increase in water consumption was noted on the 7th (p < 0.01), 14th (p < 0.05), 21st (p < 0.001), and 28th day (p < 0.001) of the experiment, compared to the normal control group (Fig. 1C). Compared to the diabetic control group, glibenclamide and AERS at 200 mg/kg resulted in a decrease in water intake on the 7th day (p < 0.05) of the experiment. Only the AERS at 200 mg/kg significantly reduced water consumption on day 14. On days 21 and 28, the decrease in water consumption was observed in rats receiving glibenclamide (p < 0.001) and AERS at 50 (p < 0.05), 100 (p < 0.01), and 200 mg/kg (p < 0.001).

### 3.3 Effects of AERS on body weight, water intake, and food consumption in T2D animals

**Fig. 2:**
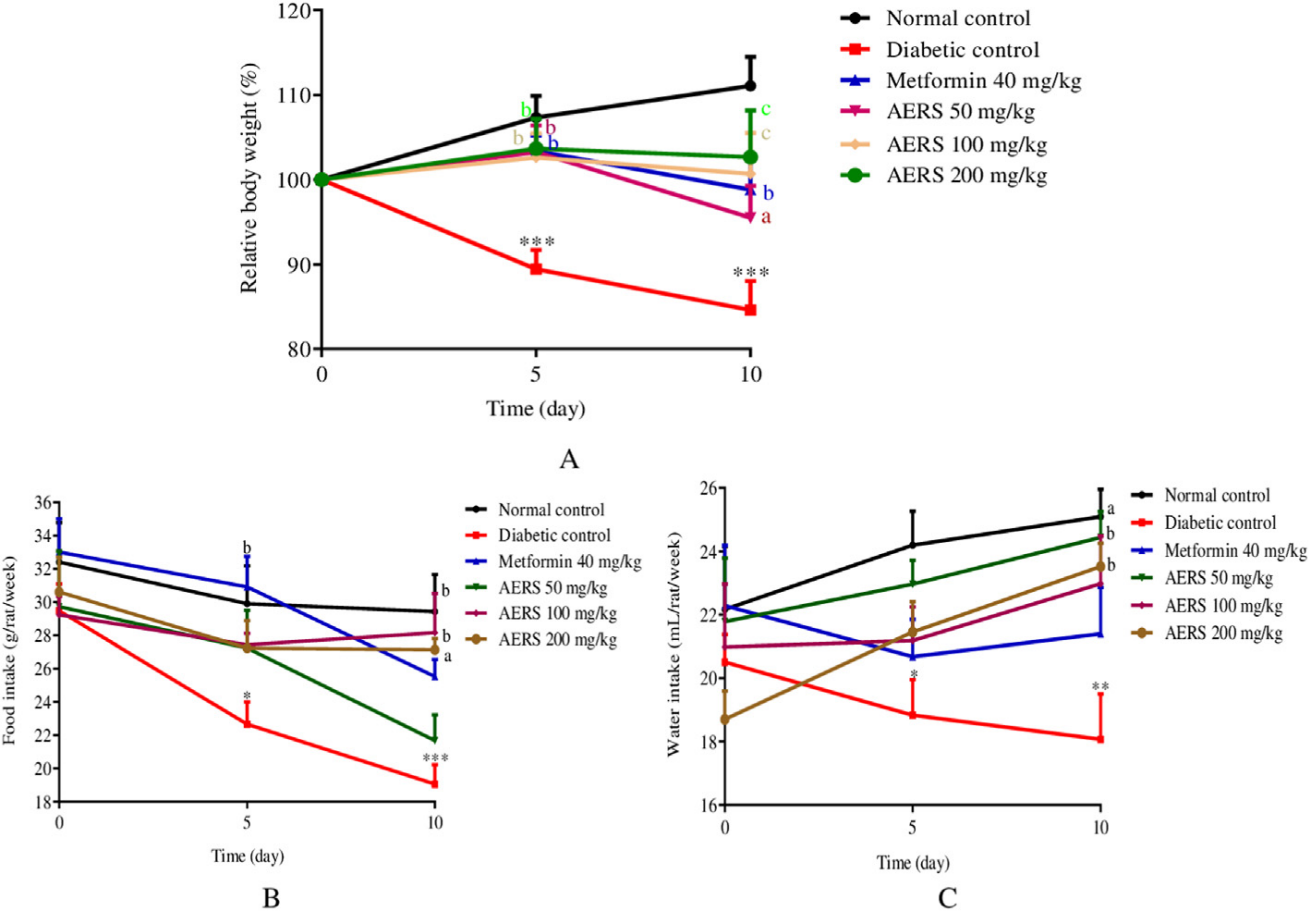
Effects of AERS (50, 100, 200 mg/kg) and metformin (40 mg/kg) on relative body weight (A), food consumption (B), water intake (C) in dexamethasone-induced T2D rats. Data were expressed in mean ± S.E.M. (n = 5). ∗p < 0.05; ∗∗p < 0.01; ∗∗∗p < 0.001 compared to the normal control group. ^a^p < 0.05; ^b^p < 0.01; ^c^p < 0.001 compared to the diabetic control group.

Compared to the normal control group, there was a significant (p < 0.001) decrease in the relative body weight of the animals in the diabetic control group. On the other hand, there is a significant increase in the relative body weight of T2D animals receiving metformin (p < 0.01) and AERS at 50 (p < 0.01), 100 (p < 0.001), and 200 mg/kg (p < 0.001), compared to the diabetic control group (Fig. 2A).

A significant decrease in food intake was observed in rats in the diabetic control group on days 5 (p < 0.05) and 10 (p < 0.001) of treatment, compared to the normal control group. Compared to the diabetic control group, animals receiving metformin showed a significant (p < 0.01) increase in food intake on the 5th (p < 0.01) and 10th day (p < 0.05) of the experience. This increase was greater (p < 0.01) on day 10 of the experiment with AERS at doses of 100 and 200 mg/kg (Fig. 2B).

On the first day of treatment, the water consumption of all experimental animals is similar (Fig. 2C). On the other hand, a significant reduction in water consumption was observed on days 5 (p < 0.05) and 10 (p < 0.01) in the animals of the diabetic control group, compared to the normal control group. On the 10th day of treatment, water consumption significantly increased with metformin (p < 0.05) and AERS (p < 0.01) at doses of 100 and 200 mg/kg, compared to the sick control group.

### 3.4 Effects of AERS on blood glucose, insulinemia, HOMA-IR, and HOMA-β of T1D rats

**Fig. 3:**
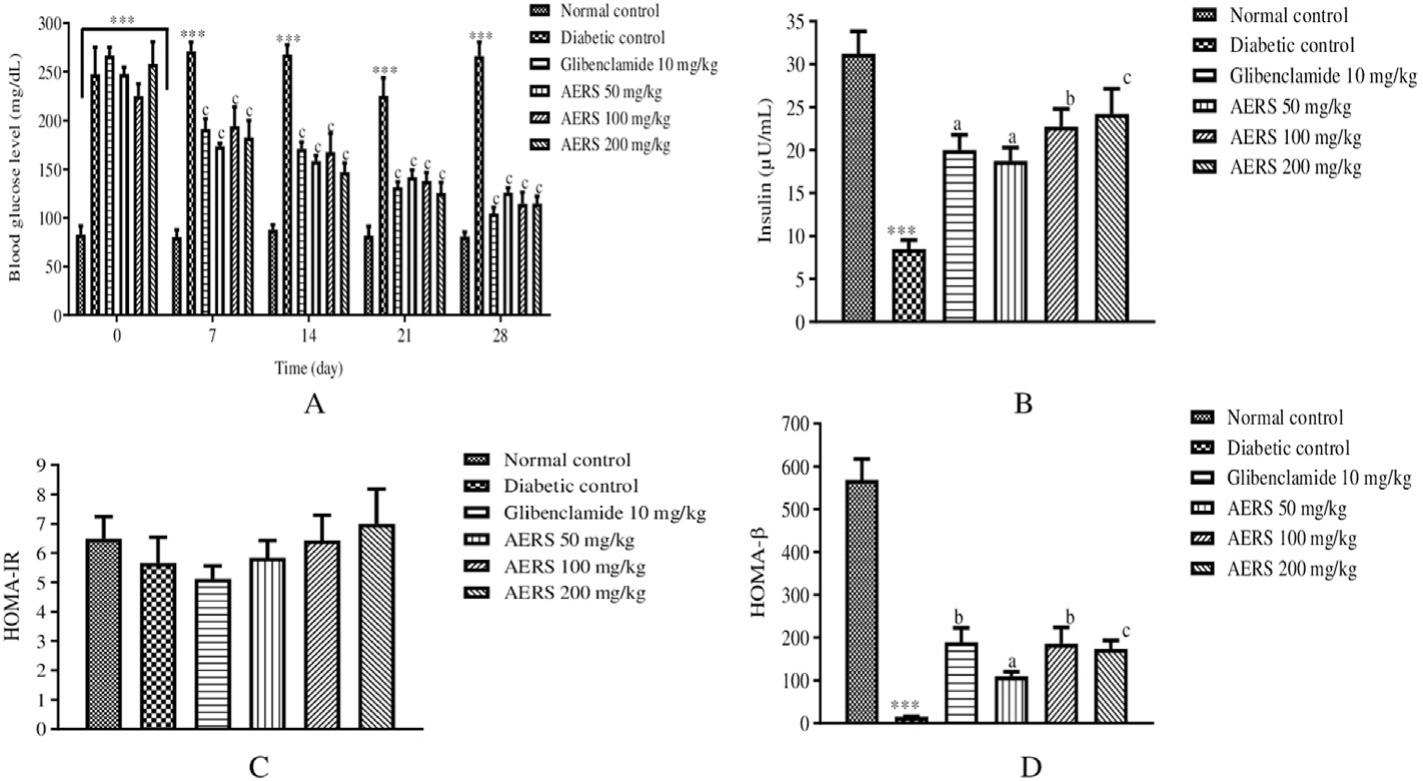
Effects of AERS (50, 100, 200 mg/kg) and glibenclamide (10 mg/kg) on blood glucose level (A), insulin level (B), HOMA-IR (C), HOMA-β (D) in streptozotocin-induced T1D rats. Data were expressed in mean ± S.E.M. (n = 5). ∗∗∗p < 0.001 compared to the normal control group. ^a^p < 0.05; ^b^p < 0.01; ^c^p < 0.001 compared to the diabetic control group.

On the 1st day of the experiment, glycaemia in T1D animals was elevated (p < 0.001) compared to that of rats in the normal control group. However, in the diabetic control group, fasting blood glucose remained high (p < 0.001) throughout the treatment period, compared to the normal control. On the other hand, in the rats receiving the reference product and the different doses of AERS, blood glucose significantly decreased (p < 0.001) throughout the treatment, compared to untreated T1D rats (Fig. 3A).

Compared to the normal control, the insulin level was reduced (p < 0.001) in the serum of rats in the diabetic control group. On the other hand, an important and dose-dependent increase in serum insulin level was observed with glibenclamide (p < 0.05) and AERS at 50 (p < 0.05), 100 (p < 0.01), and 200 mg/kg (p < 0.001), compared to untreated T1D rats (Fig. 3B).

HOMA-IR values did not undergo any significant variation in all groups of animals while HOMA-β significantly decreased (p < 0.001) in the sick control group, compared to the normal control group. In addition, an increase in HOMA-β values were observed in T1D rats receiving glibenclamide (p < 0.01) and AERS at 50 (p < 0.05), 100 (p < 0.01), and 200 mg/kg (p < 0.001), compared to the sick control (Fig. 3C).

### 3.5 Effects of AERS on blood glucose, insulinemia, HOMA-IR, and HOMA-β of T2D rats

**Fig. 4:**
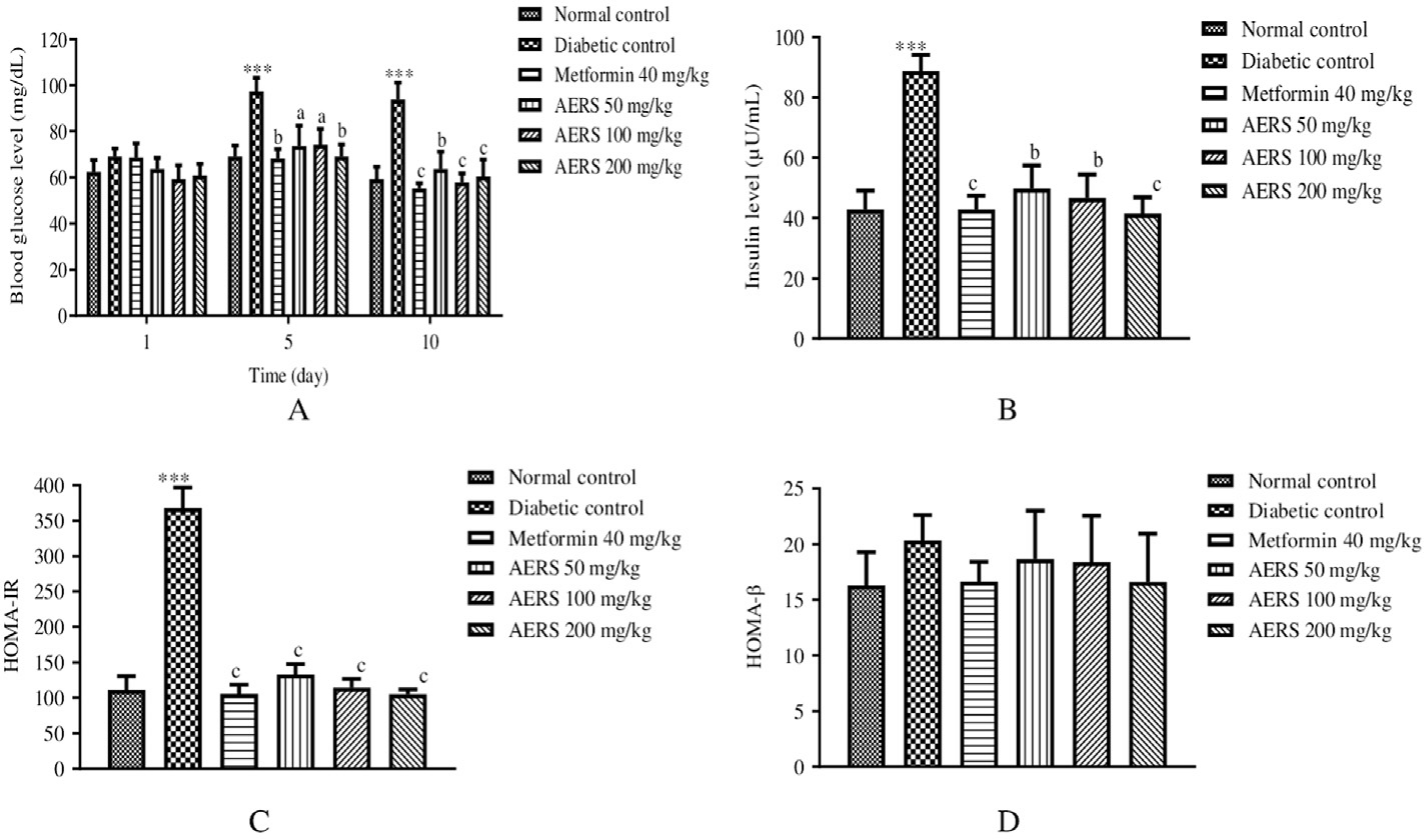
Effects of AERS (50, 100, 200 mg/kg) and metformin (40 mg/kg) on blood glucose level (A), insulin level (B), HOMA-IR (C), HOMA-β (D) in dexamethasone-induced T2D rats. Data were expressed in mean ± S.E.M. (n = 5). ∗∗∗p < 0.001 compared to the normal control group. ^a^p < 0.05; ^b^p < 0.01; ^c^p < 0.001 compared to the diabetic control group.

Fasting blood glucose in all animal groups was almost similar on day 1 of the experiment. Compared to untreated T2D rats, on days 5 and 10 of treatment, there was a significant increase (p < 0.001) in glycaemia in the diabetic control group. On the other hand, on days 5 and 10 of treatment, blood glucose significantly decreased in the animals given metformin (p < 0.001) and AERS at 50 (p < 0.01), 100 (p < 0.001), and 200 mg/kg (p < 0.001), compared to the sick control (Fig. 4A).

**Fig. 5:**
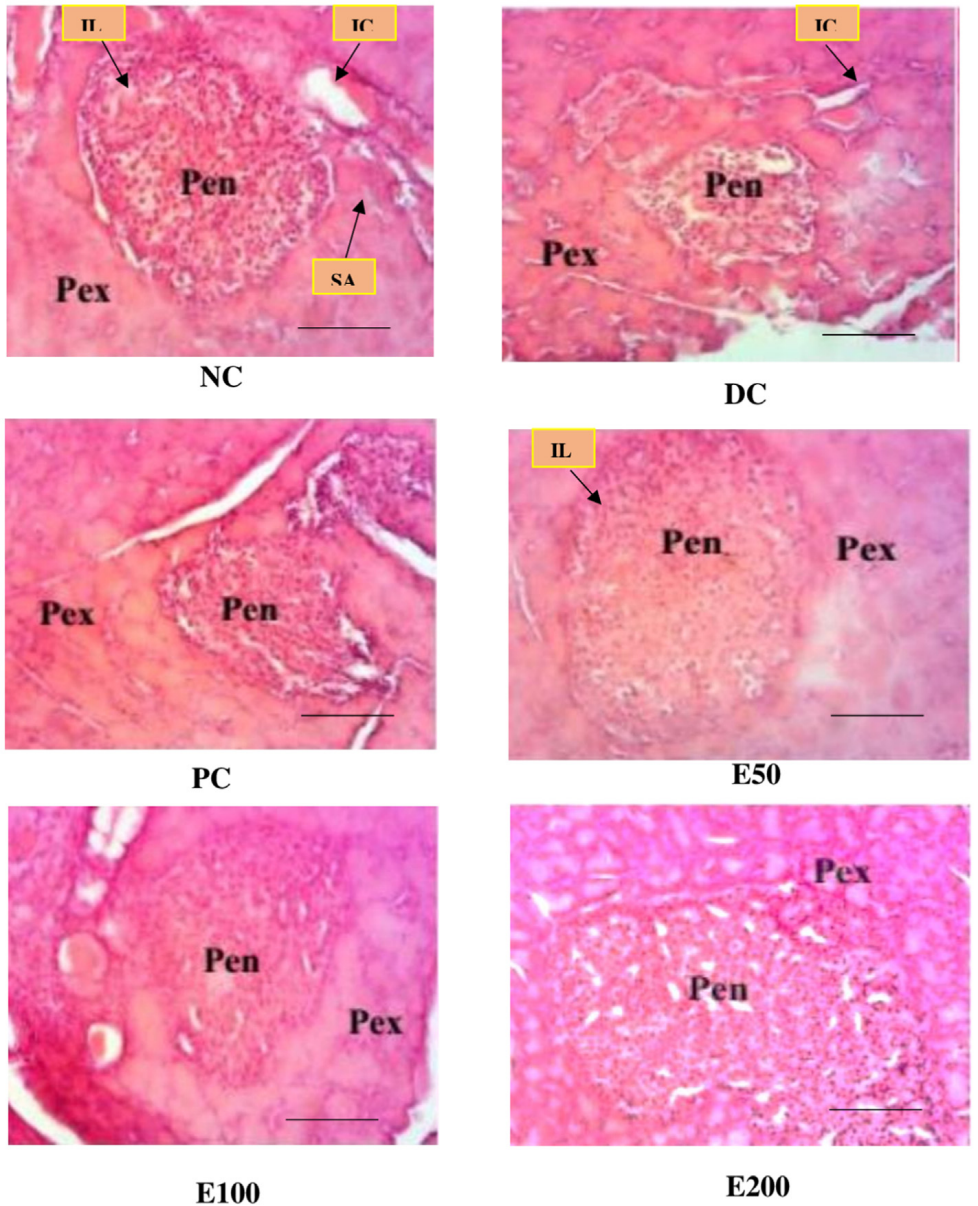
Photograph of the microarchitecture of the pancreas of T1D rats treated with AERS (50, 100, 200 mg/kg) and glibenclamide (10 mg/kg) for 28 days (H&E X 100, scale bar 50 μm). NC = normal control group, DC = negative control group, PC = positive control group, E50 = AERS 50 mg/kg, E100 = AERS 100 mg/kg, E200 = AERS 200 mg/kg, Pen = endocrine pancreas, Pex = exocrine pancreas, IL: islets of Langerhans, SA: secretory acini, IC: interlobular ducts.

Daily administration of dexamethasone resulted in an increase (p < 0.001) in insulin levels, compared to the normal control group. In contrast, the insulin level of T2D rats receiving metformin and AERS at various doses was reduced (p < 0.001), compared to the disease control group (Fig. 4B). Compared to the normal control group, HOMA-IR values increased (p < 0.001) in the diabetic control group. In animals groups treated with metformin and AERS, there was a decrease (p < 0.001) in HOMA-IR values, compared to the diabetic control group. However, there is no significant variation in HOMA-β values in any group of animals (Fig. 4D).

### 3.6 Effects of AERS on biochemical parameters and cardiovascular indices of T1D rats

Compared to the normal control group, there was an increase (p < 0.01) in the levels of TG, TC, LDL-c, VLDL-c, and LDL-c and a significant (p < 0.01) drop in HDL-c level, compared to the diabetic control group. On the other hand, in animals given glibenclamide and various doses of AERS, a drop (p < 0.01) in TC, TG, VLDL-c, and LDL-c levels and a significant (p < 0.01) increase in HDL-c level were observed, compared to the disease control group (Table 1).

**Table 1:** Effects of AERS on biochemical parameters and cardiovascular indices of T1D rats.

Groups	Normal control	Diabetic control	Glibenclamide 10 mg/kg	AERS 50 mg/kg	AERS 100 mg/kg	AERS 150 mg/kg
CT (mg/dL)	78.77 ± 8.08	133.02 ± 7.71∗∗∗	94.55 ± 8.12a	105.26 ± 6.57	92.98 ± 8.07a	87.75 ± 8.00b
TG (mg/dL)	93.56 ± 8.53	155.21 ± 10.45∗∗	102.01 ± 9.84b	111.41 ± 7.86a	97.29 ± 9.18b	101.85 ± 11.49b
LDL-c (mg/dL)	16.03 ± 5.12	79.47 ± 8.73∗∗∗	28.90 ± 4.43c	40.55 ± 5.46c	24.94 ± 4.04c	26.12 ± 4.75c
VLDL (mg/dL)	18.71 ± 1.70	31.04 ± 2.09∗∗	20.40 ± 1.96b	22.28 ± 1.57a	19.45 ± 1.83b	20.37 ± 2.29b
HDL-c (mg/dL)	50.21 ± 4.73	22.50 ± 2.80∗∗	45.24 ± 3.58a	42.42 ± 5.67a	48.58 ± 5.42b	50.06 ± 3.16b
MDA (mg/dL)	20.34 ± 2.63	31.22 ± 2.64∗∗	21.16 ± 2.12a	18.56 ± 1.18b	15.814 ± 1.13c	15.81 ± 1.13c
CAT (U/mL)	61.74 ± 4.23	31.66 ± 2.80∗∗∗	54.66 ± 2.83b	47.66 ± 5.18	55.06 ± 2.60b	55.06 ± 2.60b
SOD (U/mL)	15.70 ± 1.39	5.98 ± 0.52∗∗	14.61 ± 1.92a	12.86 ± 0.91	14.28 ± 2.00a	14.28 ± 2.00a
GSH (mg/dL)	29.14 ± 1.58	13.66 ± 1.33∗∗∗	27.86 ± 3.15b	25.66 ± 1.63b	28.06 ± 1.80b	28.06 ± 1.80b
IAA	1.49 ± 0.29	0.20 ± 0.03	0.97 ± 0.12	0.71 ± 0.12	1.22 ± 0.20	3.31 ± 2.21
IRC	1.62 ± 0.20	6.25 ± 0.75∗∗∗	2.09 ± 0.13c	2.63 ± 0.35c	1.98 ± 0.26c	1.79 ± 0.21c
IC	0.92 ± 0.07	0.31 ± 0.04∗∗∗	1.70 ± 0.25c	2.93 ± 0.22c	1.76 ± 0.18c	3.06 ± 0.25c

Each value represents mean ± SEM, n = 5. Data analysis was performed by two-way ANOVA followed by Bonferroni's post-hoc test. ∗∗p < 0.01; ∗∗∗p < 0.001 compared to the normal control. ap < 0.05; bp < 0.01; cp < 0.001 compared to the diabetic control.

In untreated T1D animals, MDA level increased (p < 0.01) while GHS level (p < 0.01), and SOD and CAT activities (p < 0.001) were significantly decreased, compared to untreated T1D rats. However, compared to the sick control group, the MDA level was significantly reduced in the rats of the standard group (p < 0.05) and those receiving AERS (p < 0.01). Furthermore, GSH level and CAT activity increased (p < 0.01) in animals groups receiving the reference product and various doses of AERS. SOD activity was also decreased (p < 0.05) with glibenclamide and AERS at doses of 100 and 200 mg/kg (Table 1).

Compared to untreated T1D animals, no significant change in IAA value was observed while IRC and IC values increased (p < 0.001) in the sick control group (Table 1). Moreover, compared to the diabetic control group, a significant decrease (p < 0.01) in IRC value and an increase (p < 0.001) in IC value were observed with glibenclamide and various doses of AERS.

### 3.7 Effects of AERS on biochemical parameters and cardiovascular indices of T2D rats

Table 2 shows the effects of AERS on lipid profile, oxidative stress parameters, and cardiovascular indices of T2D animals. It emerges from this table that compared to the normal control group, there is an increase (p < 0.001) in TC, TG, VLDL-c, and LDL-c levels, and a decrease (p < 0.001) in HDL-c level in untreated T2D rats. On the other hand, AERS at various doses and metformin caused a significant (p < 0.001) decrease in LDL-c and CT levels, and an increase (p < 0.001) in HDL-c level, compared to the sick control group. Likewise, an important decrease in TG and VLDL-c levels was noted with metformin (p < 0.05) and AERS at 50 (p < 0.01), 100 (p < 0.01), and 200 mg/kg (p < 0.001).

**Table 2:** Effects of AERS on biochemical parameters and cardiovascular indices of T2D rats.

Groups	Normal control	Diabetic control	Dexa + Met 40 mg/kg	Dexa + AERS 50 mg/kg	Dexa + AERS 100 mg/kg	Dexa + AERS 200 mg/kg
CT (mg/dL)	111.58 ± 5.121	142.61 ± 4.36∗∗∗	100.17 ± 2.61^c^	96.73 ± 1.72^c^	93.21 ± 4.37^c^	85.96 ± 3.22^c^
TG (mg/dL)	67.26 ± 3.80	119.76 ± 4.58∗∗∗	98.37 ± 4.07^a^	93.12 ± 5.05^b^	89.95 ± 4.22^b^	80.15 ± 5.028^c^
LDL-c (mg/dL)	51.63 ± 4.60	90.98 ± 5.93∗∗∗	33.54 ± 5.48^c^	21.49 ± 3.11^c^	27.76 ± 2.58^c^	19.61 ± 3.39^c^
VLDL (mg/dL)	13.45 ± 0.76	23.95 ± 0.91∗∗∗	19.67 ± 0.81^a^	18.62 ± 1.01^b^	17.99 ± 0.84^b^	16.03 ± 1.00^c^
HDL-c (mg/dL)	46.50 ± 2.36	27.67 ± 2.22∗∗∗	46.95 ± 3.83^c^	56.62 ± 2.05^c^	47.46 ± 2.41^c^	50.31 ± 1.15^c^
MDA (mg/mL)	12.08 ± 1.01	20.48 ± 2.619∗	13.44 ± 1.79^a^	11.28 ± 1.48^b^	10.66 ± 0.89^b^	9.44 ± 0.76^c^
CAT (U/L)	12.02 ± 0.88	7.2 ± 0.88∗	10.8 ± 0.90	9.08 ± 1.17	11.66 ± 0.42^a^	12.66 ± 1.22^b^
SOD (U/L)	30.08 ± 2.29	15.32 ± 1.28∗∗	31.62 ± 2.58^b^	26.88 ± 1.77^a^	31.62 ± 2.45^b^	32.42 ± 3.66^c^
GSH (mg/dL)	8.24 ± 0.77	5.22 ± 0.49	7.8 ± 0.67	8.42 ± 0.73	9.04 ± 0.81^a^	9.28 ± 1.06^a^
IAA	2.57 ± 0.46	0.30 ± 0.02∗∗∗	0.98 ± 0.17	1.77 ± 0.34a	1.25 ± 0.27	1.85 ± 0.28a
IRC	2.41 ± 0.11	5.29 ± 0.45∗∗∗	2.20 ± 0.21c	1.72 ± 0.08c	1.97 ± 0.07c	1.71 ± 0.06c
IC	0.92 ± 0.07	0.31 ± 0.04	1.70 ± 0.47	1.76 ± 0.18	2.93 ± 0.52b	3. 06 ± 0.76b

Each value represents the mean ± SEM; n = 5. ∗p < 0.05; ∗∗p < 0.01; ∗∗∗p < 0.001 statistically significant difference from the normal control group. ap < 0.05; bp < 0.01; cp < 0.001 statistically significant compared to the diabetic control.

Compared to the normal control group, animals in the sick control group showed an increase (p < 0.05) in MDA level, and a drop in CAT (p < 0.05) and SOD (p < 0.01) activities. On the other hand, compared to the diabetic control group, there was a significant decrease in MDA level with metformin (p < 0.05) and AERS at 50 (p < 0.01), 100 (p < 0.01), and 200 mg/kg (p < 0.001). Likewise, SOD activity was significantly reduced with metformin (p < 0.001) and AERS at 50 (p < 0.05), 100 (p < 0.01), and 200 mg/kg (p < 0.001). AERS (100 and 200 mg/kg) induced a significant (p < 0.05; p < 0.01) decrease in CAT activity. GSH level did not vary significantly in all groups of animals. (Table 2).

In the animals of the diabetic control group, a significant (p < 0.01) decrease of IAA values and an increase (p < 0.01) in CRI values were noted, compared to the normal control group. On the other hand, AERS (50, 100, and 200 mg/kg) and metformin induced a significant (p < 0.001) decrease in CRI values. AERS (100 and 200 mg/kg) induced a drop (p < 0.01) in CI values (Table 2).

### 3.8 Histological sections of pancreas in T1D rats

The results of the histological analysis of the pancreas of the diabetic rats treated with AERS are shown in Fig. 5. The analysis of the pancreas of the animals of the normal control group (NC) shows a normal architecture presenting an exocrine pancreas (Pex) with secretory acini (SA) and interlobular ducts (IC), and an endocrine pancreas (Pen) composed of islets of Langerhans (IL). However, in the animals of the negative control batch (DC), a reduction in the size and number of the pancreatic islets is observed, compared with the neutral control group. Administration of glibenclamide (PC) and AERS at doses of 50 (E50), 100 (E100) and 200 mg/kg (E200) resulted in an increase in the size and number of islets of Langerhans, compared to animals from the diabetic control group.

## 4 Discussion

Insulin resistance precedes T2D; one of the mechanisms used to delay the development of diabetes and its complications is the reduction of insulin resistance. Dexamethasone induces insulin resistance by inhibiting the action of insulin; that is, by stimulating the production of glucose in the liver and reducing the use of glucose in the peripheral tissues.^27^ Antidiabetic drugs so far used in conventional medicine are of synthetic origin, have many side effects and are not always available.^9^ Treatment with natural plants is the remedy used by local populations and recommended by WHO. The present study aimed to evaluate the antidiabetic properties of AERS in T1D and T2D induced by STZ and dexamethasone respectively.

Dexamethasone stimulates the production of myostatin resulting in muscle atrophy and reduced body weight in animals.^28^ It has a strong ability to inhibit the transport of glucose into cells, resulting in lack of fats due to insulin resistance.^29^ STZ is a selective beta cell cytotoxic agent that destroys these cells, resulting in decreased insulin secretion. Insufficient insulin in diabetics causes weight loss by reducing lipid synthesis.^30^ In the present study, the administration of dexamethasone and STZ resulted in body weight loss in animals. However, in animals receiving reference drugs and AERS, a significant increase in body weight was noted. These results are similar to those obtained previously by Mahamad et al.^31^ who showed that *Cissus polyantha* caused an increase in relative body weight of diabetic animals. Diabetes is accompanied by an increase in glycogenolysis, lipolysis, and gluconeogenesis; these biochemical activities lead to muscle wasting and loss of tissue proteins. AERS is believed to prevent these changes and thereby restore body weight in diabetic treated rats.^32^

Polyphagia and polydipsia are symptoms of diabetes mellitus that are caused by insulin deficiency or the lack of insulin utilization by target organs. These parameters essentially tell us about the reestablishment of insulin secretion as well as the degree of glucose utilization by the cells. The exaggeration of food consumption indicates that the body cannot use glucose, although it is abundantly provided that it draws from its reserves of lipids and proteins for its energy metabolism.^33^ The increase in water consumption in diabetics is caused by hyperglycemia that overflows in the urine leading to dehydration. In the present study, administration of standard products and AERS (100 and/or 200 mg/kg) resulted in reduced polyphagia and polydipsia in diabetic animals. These findings suggest that AERS affects the neuroendocrine regulation of food intake by the digestive system, including nutrient sensing and peptide secretion by enteroendocrine cells. Since the centers that control food and water intake are located in the hypothalamus, it is likely that AERS affects these centers. Indeed, food intake suppression is a function of the satiety center in the ventromedial hypothalamus and water intake is influenced by osmoregulators in the anterior hypothalamus that sense the osmolality of body fluids. Therefore, AERS could stimulate the satiety center while inhibiting the thirst center (osmoreceptors) as well as the hunger center (lateral hypothalamic nuclei).^34^

Dexamethasone causes hyperglycemia by two mechanisms, on the one hand by an overproduction mechanism (excess of gluconeogenesis and glycogenolysis) on the other hand by the decrease in the use of glucose by the peripheral tissues.^35^ STZ causes the destruction of beta cells in the islets of Langerhans in the pancreas resulting in an increase in blood glucose levels.^36^ In this study, dexamethasone and STZ caused an increase in fasting glycaemia in experimental animals. However, the administration of AERS and reference products induced a decrease in glycaemia in diabetic animals. Miaffo et al.^37^, a, b obtained similar results with *Guiera senegalensis* extract. Metformin is an antidiabetic agent that acts by inhibiting glycogenolysis, reducing glucose absorption from the gastrointestinal tract, and stimulating the absorption and peripheral utilization of glucose.^38^ AERS may also act like metformin to lower blood sugar. The decrease in blood glucose level after treatment could be due to the ability of AERS to enhance the translocation of GLUT 4 from the inner membrane to the plasma membrane of muscular cells accompanied by a change in the total amount of GLUT4 or its gene expression.^38^ On the other hand, glibenclamide is an antidiabetic agent which stimulates the β-cells of the pancreas to secrete insulin. AERS would act like glibenclamide by inhibiting ATP-sensitive K+ channels and causing insulin secretion. The closure of the K+ channel, caused by the increase in the concentration of ATP, leads to a depolarization of the plasma membrane responsible for the opening of voltage-gated Ca2+ channels and therefore an increase in intracellular free Ca2+. Ca2+ allows the binding of secretory vesicles containing insulin to the plasma membrane of β-pancreatic cells, and consequently the secretion of insulin by exocytosis.^39^ Phenolic compounds may participate in the regulation of carbohydrate metabolism by causing intestinal glucose absorption, insulin secretion, and stimulation of insulin receptor activity in insulin sensitive tissues.^40^^,^^41^ The regulation of glucose and insulin levels observed in this work would be linked to these secondary metabolites (flavonoids, terpenoids, and glycosides) present in the AERS.

In diabetes, insulin deficiency or insulin resistance is accompanied with dyslipidemia characterized by increase in VLDL-c, TG, TC, and LDL-c levels and decrease in HDL-c level.^42^ In this work, administration of dexamethasone and/or STZ to experimental animals results in dyslipidemia. However, standard products and AERS prevented this dyslipidemia. AERS would have acted by activating the oxidation of fatty acids and inhibits lipogenesis enzymes by activating AMPK and helps lower blood triglyceride levels.^43^ AERS may inhibit the activity of key enzymes in cholesterol biosynthesis and decrease the ability of LDL to transport free cholesterol to tissues.^44^ The increase in HDL-c levels may be due to the power of AERS to restore insulin secretion because insulin would increase the activity of Lecithin Cholesterol Acyl Transferase which will be at the origin of the formation of HDL-c molecules.^45^ The increase in this cardioprotective lipid (HDL-c) suggests a possible preventive effect of AERS against the formation of arteriosclerosis. This is further justified by the increase in IAA and CI values, and the decrease in CRI value observed in the present work. Limaye et al.^46^ showed that bioactive compounds such as flavonoids and phenols have hypolipidemic activity. The antidyslipidemic effect could be due to the presence of these secondary metabolites in the AERS.

Oxidative damage plays a major role in the progressive development of diabetes mellitus.^47^ In the diabetic state, the various pathways of the generation of free radicals are among others increased glycolysis, intercellular activation via the polyols, auto-oxidation of glucose, and non-enzymatic glycation of protein.^48^ In this study, an increase in MDA level and a decrease in antioxidant enzymes (CAT and SOD) activities and GSH levels were noted in diabetic rats. However, standard products (glibenclamide, metformin) and/or EARS prevented or restored these oxidative damages. AERS would have prevented oxidative damage by trapping and cleaning free radicals in order to restore the antioxidant parameters disturbed by STZ and dexamethasone. According to Patel et al.,^49^ phenols and flavonoids have antioxidant properties. These compounds possess the antioxidant potential via their reducing power or their potential for donating or transferring electrons or hydrogen.^50^ This indicated an improvement in the β cells of the pancreas as shown by the results of the histological section where an increase in the number and size of the islets of Langerhans of the pancreas of diabetic animals treated with standard products and AERS was observed.

## 5 Conclusion

In short, AERS exhibits hypoglycemic, lipid-lowering, cardioprotective, and antioxidant activities. This antidiabetic potential is believed to be due to the bioactive constituents present in AERS. These results justify the traditional use of AERS for the management of diabetes and its complications. In addition, more work is needed to elucidate the probable cellular and molecular mechanisms of action of AERS.

## Authors contributions statement

David Miaffo and Fidèle Ntchapda helped design the study and provided the reagents for the different assays. Barthelemy Maidadi proposed the plant material. Barthelemy Maidadi and Abba Talba Mahamad harvested the plant material and prepared the crude extract. Barthelemy Maidadi, David Miaffo, and Abba Talba Mahamad conducted the experiments. David Miaffo analyzed the data. Barthelemy Maidadi and David Miaffo drafted the article. Fidèle Ntchapda corrected the final version of the document and all the authors approved it.

## Funding

This research did not receive any grant from any funding agencies in public, commercial or non-proﬁt sector.

## Declaration of competing interest

The authors declare that they have no conﬂicts of interest related to the publication of this study.
